# Bead-linked transposomes enable a normalization-free workflow for NGS library preparation

**DOI:** 10.1186/s12864-018-5096-9

**Published:** 2018-10-01

**Authors:** Stephen Bruinsma, Joshua Burgess, Daniel Schlingman, Agata Czyz, Natalie Morrell, Catherine Ballenger, Heather Meinholz, Lee Brady, Anupama Khanna, Lindsay Freeberg, Rosamond G Jackson, Pascale Mathonet, Susan C Verity, Andrew F Slatter, Rooz Golshani, Haiying Grunenwald, Gary P Schroth, Niall A Gormley

**Affiliations:** 1 Illumina, Inc., Madison, WI USA; 2 Illumina, Inc., Great Abington, Cambridge, UK; 3 Illumina, Inc., San Diego, California USA

**Keywords:** Next-generation sequencing, Library preparation, Transposome

## Abstract

**Background:**

Transposome-based technologies have enabled the streamlined production of sequencer-ready DNA libraries; however, current methods are highly sensitive to the amount and quality of input nucleic acid.

**Results:**

We describe a new library preparation technology (Nextera DNA Flex) that utilizes a known concentration of transposomes conjugated directly to beads to bind a fixed amount of DNA, and enables direct input of blood and saliva using an integrated extraction protocol. We further report results from libraries generated outside the standard parameters of the workflow, highlighting novel applications for Nextera DNA Flex, including human genome builds and variant calling from below 1 ng DNA input, customization of insert size, and preparation of libraries from short fragments and severely degraded FFPE samples. Using this bead-linked library preparation method, library yield saturation was observed at an input amount of 100 ng. Preparation of libraries from a range of species with varying GC levels demonstrated uniform coverage of small genomes. For large and complex genomes, coverage across the genome, including difficult regions, was improved compared with other library preparation methods. Libraries were successfully generated from amplicons of varying sizes (from 50 bp to 11 kb), however, a decrease in efficiency was observed for amplicons smaller than 250 bp. This library preparation method was also compatible with poor-quality DNA samples, with sequenceable libraries prepared from formalin-fixed paraffin-embedded samples with varying levels of degradation.

**Conclusions:**

In contrast to solution-based library preparation, this bead-based technology produces a normalized, sequencing-ready library for a wide range of DNA input types and amounts, largely obviating the need for DNA quantitation. The robustness of this bead-based library preparation kit and flexibility of input DNA facilitates application across a wide range of fields.

## Background

Library preparation is an important first step for all next-generation sequencing (NGS) applications and generally employs several common steps. First, the DNA is fragmented, by mechanical or enzymatic means, before end-repair. Next, DNA fragments undergo 5′ and 3′ ligation of platform-specific adapters and PCR amplification [1]. Recently, in vitro transposition has been used to generate sequencer-ready libraries from genomic DNA (gDNA) [2] with dramatic time savings and reduced input requirements. Transposon-based library construction improves the DNA library preparation process by eliminating the need for a separate DNA fragmentation step and removing the prerequisite for ligation between DNA fragments [3]. The efficiency of fragmentation is highly dependent on the enzyme to DNA ratio. Thus, variable DNA input amounts will generate inconsistencies in the fragment size distribution of the library. This introduces variability in the library preparation process that can have downstream effects on sequencing coverage.

Herein, we describe a new library preparation technology, which employs magnetic-bead linked transposomes (BLT). Using a known quantity of transposomes conjugated directly to beads fixes the amount of DNA (for inputs of at least 100 ng), allowing libraries with consistent fragment sizes and yields to be generated in a few hours. We have optimized the methodology to maximize library diversity, which results in improved coverage uniformity across the genome. We illustrate the broad applicability of this method, from amplicons and small genomes to large or complex genomes.

## Results

### Normalized yields and index representations

**Fig. 1:**
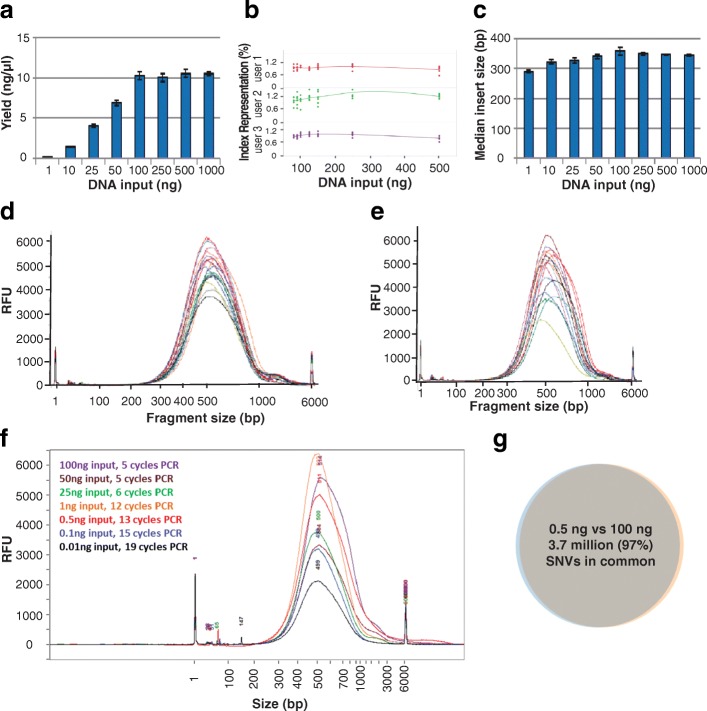
Bead-based tagmentation reduces insert size variation and normalizes yield. Libraries were prepared from human gDNA (NA12878). **a** Library yield was determined by Qubit and was directly correlated with DNA input amount for inputs smaller than 100 ng; the beads became saturated at 100 ng leading to normalized yields of around 11 ng/μl. Sequencing of the libraries on a MiSeq system and data analysis by the BaseSpace Whole Genome Sequencing app revealed consistent index representation across different DNA input amounts for multiple users (**b**) and a median insert length that was slightly smaller at lower DNA input amounts but was consistently around 350 bp for sample inputs of 100 ng and above (**c**). Libraries prepared from integrated DNA extraction protocols for blood (**d**) and saliva (**e**). **f** Library traces for low DNA input amounts. **g** There was almost complete overlap in single nucleotide variant (SNV) calls for libraries prepared from 0.5 ng and 100 ng inputs; each library was sequenced on one lane of a HiSeqX with data analysis performed using the BaseSpace Whole Genome Sequencing app and Variant Calling Assessment Tool

We theorized that with bead-linked transposome methodology, because a given number of transposomes are conjugated directly to beads at a fixed spacing, the beads will bind a fixed maximum amount of DNA. In binding a fixed amount of DNA, the immobilized transposomes should fragment the DNA to a set size distribution, leading to library yield normalization at a saturating DNA input amount. Consistent yields and fragment sizes should be achieved above this saturation point. To test this, we evaluated yields for varying amounts of input DNA. We determined that the beads become saturated at an input amount of around 100 ng, with normalized yields of around 330 ng observed for inputs of 100 ng to 1 μg (Fig. 1a); for DNA inputs less than 100 ng, the library yield was directly correlated to the input amount. Pooling equal volumes of 105 libraries without manual normalization resulted in consistent index representation (average coefficient of variation of 14.9% across users) across multiple users for inputs of 100 to 500 ng suggesting a normalized quantity of each library was generated (Fig. 1b). As such, for input amounts of 100 ng or greater, DNA quantitation is unnecessary to achieve normalization. To further test the ability of Nextera DNA Flex to enable quantitation-free normalization, blood and saliva samples were collected, DNA isolated with the integrated extraction workflow, and libraries prepared without prior quantification of DNA amount. Library yields and fragment size distributions (Fig. 1d and e) were consistent with that obtained from 100 ng purified DNA, confirming saturation and normalization of library yield without quantification. We observed that the mean insert length was slightly smaller at lower DNA input amounts but was consistently around 350 bp for sample inputs of 100 ng and above (Fig. 1c). This size is optimal for sequencing at paired-end read lengths of 150 cycles. Thus, with bead-linked tagmentation, consistent insert sizes are achieved independent of DNA input amount.

To further explore the relationship of DNA input amount with Nextera DNA Flex library generation, we generated a set of libraries from human DNA (NA12878), varying the input from 0.01 ng (10 pg) to 100 ng. Libraries were successfully generated for each input amount (Table 1, Fig. 1f) by increasing PCR cycle number according to DNA input, with a minimum yield of 100 ng from the 10 pg input. All libraries showed approximately the expected size distribution (Fig. 1f). Typically, around 370 million paired-end reads give a mean coverage of approximately 30×, which was observed for 25 ng and 100 ng inputs. At lower inputs (≤ 1 ng) all reads from a single lane of the HiSeq™ X were used for analysis. Samples were aligned and variant calling at each input amount was compared to Platinum Genome NA12878 data. As low as 0.1 ng input, greater than 99% of genome bases were covered at least 1× (Table 1), with autosome callability of 73%. At 0.5 ng and above, autosome callability was above 96%, and single nucleotide variant (SNV) recall and precision were above 98%. In fact, comparing SNV calls between 0.5 ng input and 100 ng input, we found that 97% of calls were shared (Fig. 1g), demonstrating strong concordance. As expected, insertion and deletion (Indels) variant calls dropped more significantly with increased PCR cycles, probably due to polymerase slippage during PCR amplification, but 88% Indel recall with 0.5 ng input was achieved, and 85% of Indel calls made at 100 ng input were also captured with 0.5 ng input.

**Table 1:** Sequencing metrics for libraries prepared from low DNA input amounts

Input NA12878 DNA, ng	0.01	0.1	0.5	1	25	100
PCR cycles	19	15	13	12	6	5
Library yield, ng	101.0	149.1	243.3	267.6	188.5	324.2
Total paired-end reads	4.94E + 08	5.09E + 08	4.55E + 08	5.01E + 08	5.08E + 08	5.04E + 08
Bases ≥ Q30, %	88.4%	89.3%	89.8%	89.7%	89.9%	89.0%
Aligned reads, %	94.4%	95.7%	93.5%	96.3%	96.8%	96.6%
Insert size median, bp	258	255	241	302	317	343
Mean coverage, X	2.4	9.1	22.8	30.1	40.1	40.1
Autosome coverage ≥1×, %	47.3%	99.3%	99.6%	99.6%	99.6%	99.6%
Autosome coverage ≥10×, %	5.4%	38.0%	98.1%	98.6%	98.8%	98.8%
Callability, %	7.7%	73.0%	96.5%	96.8%	97.0%	97.0%
Exon callability, %	8.0%	75.1%	98.6%	98.7%	98.9%	98.9%
SNV recall, %	14.8%	85.7%	98.2%	98.7%	99.0%	99.0%
SNV precision, %	42.5%	82.2%	99.1%	99.6%	99.8%	99.8%
Indel recall, %	5.6%	55.9%	88.1%	91.6%	95.3%	95.6%
Indel precision, %	54.1%	77.5%	83.8%	88.1%	97.1%	97.6%

Libraries were prepared from 0.01 to 100 ng of human gDNA (NA12878). Data was generated on a HiSeqX (2 × 150 bp) and data analysis performed using the BaseSpace Whole Genome Sequencing app. Q scores are a sequencing quality metric, with a Q score of 30 (Q30) indicating that the probability of an incorrect base call is 1 in 1000, which equates to a base call accuracy of 99.9%. Callability describes the percentage of base calls in the data set that pass the quality metrics required for making a genotype call; base quality, alignment quality, and minimum coverage levels are considered. *SNV* Single-nucleotide variant, *Indel* Insertion/deletion

### Impact of PCR cycle number on variant calling

There are multiple factors that influence the accuracy and sensitivity of variant calling, including analysis variables such as informatic pipeline or reference sequence and experimental factors such as input amount, DNA quality, or library preparation bias, including errors introduced by PCR. The Nextera DNA Flex workflow recommends five PCR cycles for input amounts greater than or equal to 50 ng, but the typical library yield is more than what is required for most sequencing applications, suggesting that PCR cycles could be reduced if desired. To assess the impact of PCR cycle number on the quality of human genome sequencing, we prepared libraries from 100 ng of human gDNA (NA12878) but varied the number of PCR cycles from 2 to 5. After sequencing 2 × 150 cycles, coverage and callability were compared (Table 2). Coverage was around 30X for all combinations. Autosome callability was not significantly altered by the number of PCR cycles. Interestingly, we noticed a greater change in callability because of the version of the analysis pipeline used, with a notable improvement with the updated pipeline (BaseSpace™ Whole Genome Sequencing 6.0.0). Single-nucleotide variant recall was also improved with the updated BaseSpace pipeline, and not significantly affected by the number of PCR cycles. For Indels, recall and precision were both improved by reducing the number of PCR cycles and when using the updated pipeline due to the more advanced alignment and variant calling algorithms [4–7].

**Table 2:** Improving callability by reducing PCR cycles or using an improved variant calling pipeline

Analysis version	Whole Genome Application 5.0.0				Whole Genome Application 6.0.0			
PCR Cycles	2	3	4	5	2	3	4	5
Autosome mean coverage, X	30.4	30.5	30.1	30.1	30.0	30.3	31.2	31.5
Autosome callability^a^, %	95.5	95.6	95.5	95.4	96.8	96.8	96.8	96.8
Autosome coverage at 15X, %	97.6	97.7	97.6	97.6	97.8	97.9	98.0	98.0
Autosome exon coverage at 15X, %	99.4	99.4	99.3	99.3	99.4	99.4	99.4	99.4
SNV recall, %	97.0	97.0	97.0	96.9	98.8	98.8	98.8	98.8
SNV precision, %	99.9	99.9	99.8	99.8	99.8	99.8	99.8	99.8
Indel recall, %	93.2	92.5	91.4	90.2	95.0	94.9	94.7	94.4
Indel precision, %	96.6	95.9	94.5	92.9	98.0	97.9	97.6	97.2

Libraries were prepared from 100 ng of NA12878. Data was generated on a HiSeq X (2 × 151 bp) and data analysis performed using the BaseSpace Sequence Whole Genome Sequencing Application. ^a^ Callability describes the percentage of base calls in the data set that pass the quality metrics required for making a genotype call; base quality, alignment quality, and minimum coverage levels are considered. *SNV* Single-nucleotide variant, *Indel* Insertion/deletion

### Customization of library fragment size

**Fig. 2:**
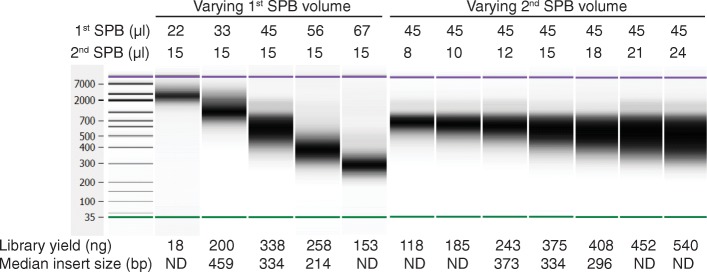
Customization of post-amplification cleanup conditions. Libraries were prepared from 100 ng of human gDNA (NA12878), sequenced on a HiSeq X, and data analysis performed using the BaseSpace Whole Genome Sequencing 6.0.0 app. The median library fragment size and yield were modified by varying the volume of the Sample Preparation Beads (SPB) used during the first or second step of post-amplification cleanup. As extreme conditions were not sequenced, the median insert size was not available for all combinations. ND, not determined

Conditions used during the post-amplification cleanup and size selection also impact the resultant library. In contrast to a single-sided solid phase reversible immobilization (SPRI) cleanup that involves one-sided size selection, double-sided solid phase reversible immobilization (SPRI) cleanup and size selection discards low (suboptimal for sequencing) and high (poorly cluster) molecular weight fragments, which reduces the yield but tightens the size distribution in the final library. We show that by adjusting the volume of sample purification beads (SPBs) used during double-sided SPRI cleanup, the final library insert size can be fine-tuned. Although for many applications, an insert size around 350 bp is optimal, for other applications different insert sizes might be preferable. To address this, we assessed the impact of adjusting the SPB volume used during the post-amplification cleanup on the library yield and fragment size distribution. The fragment size distribution was sensitive to the SPB volume, particularly during the first cleanup. As the SPB volume used during the first or second cleanup step of a double-sided protocol is increased, while keeping the volume in the other step fixed, there was a decrease in the median fragment size (Fig. 2). The yield was also impacted by the SPB volume. Consequently, a 45 μl volume of SPBs in the first cleanup step and 15 μl of SPBs in the second cleanup step was chosen for the standard protocol to produce a good yield with a median fragment size (334 bp) suitable for sequencing with 2 × 150 cycles. However, users desiring a modification in the insert size could achieve this by altering the post-amplification cleanup protocol (increasing the SPB volume to decrease fragment size, or conversely, reducing the SPB volume to increase fragment size; optimization is recommended to achieve the desired size profile) noting that the library yield may be impacted.

### Comparison to other library preparation kits

**Fig. 3:**
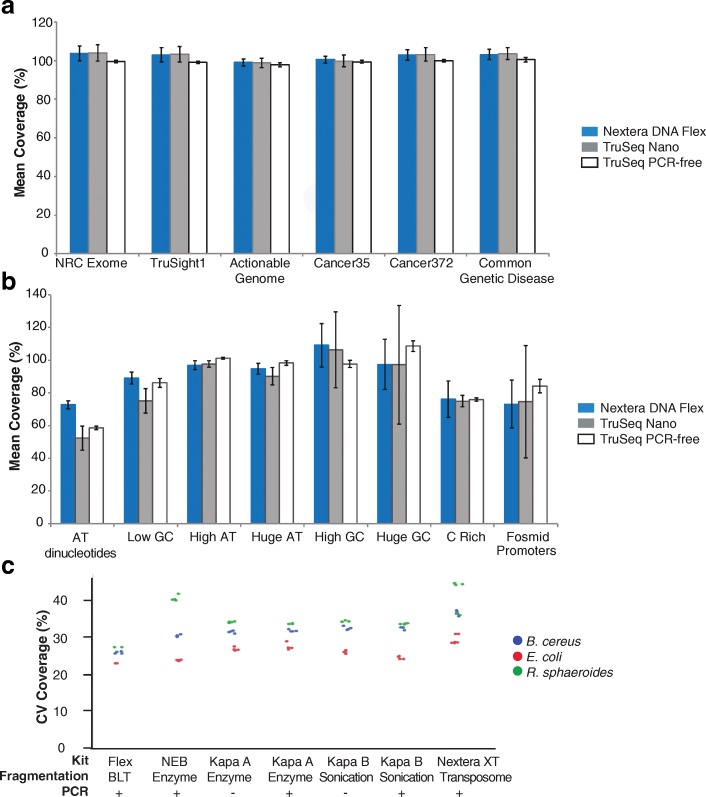
Improved coverage of human and bacterial genomes with Nextera DNA Flex. **a** Coverage across important regions of the human genome by three library preparation kits: Nextera DNA Flex, TruSeq Nano, and TruSeq PCR-free. **b** Coverage across extreme regions of the human genome by three library preparation kits: Nextera DNA Flex, TruSeq Nano, and TruSeq PCR-free. **c** Libraries generated from the small genomes of bacteria with low (*B. cereus*), medium (*E. coli*), and high (*R. sphaeroides*) GC content at 1 ng inputs. Less coverage variation was observed with libraries prepared by Nextera DNA Flex compared with libraries prepared by other commercially available library preparation kits (NEB, NEBNext Ultra DNA Library Prep Kit; Kapa A, Kapa HyperPlus Kits; Kapa B, Kapa HyperPrep Kits), particularly for the low and high GC content genomes of *B. cereus* and *R. sphaeroides.* The method of DNA fragmentation and whether PCR amplification was used during library preparation is indicated

We compared Nextera DNA Flex to a selection of popular library preparation kits by preparing libraries for 30X coverage human genome builds (Table 3). Nextera DNA Flex was the only option with integrated sample extraction from blood and saliva and normalization for inputs of at least 100 ng. Nextera DNA Flex had the fastest total assay time, a result of removing a separate DNA fragmentation step and elimination of time needed for library quantitation and normalization before pooling. Nextera DNA Flex produced sequencing results comparable to or better than the other library preparation kits. Coverage of important regions of the genome was comparable between Nextera DNA Flex libraries and libraries prepared using mechanical fragmentation-based library preparation kits, TruSeq™ Nano and TruSeq PCR-free (Fig. 3a). Coverage of some extreme regions of the genome, including stretches of AT dinucleotides and low GC regions, was improved with Nextera DNA Flex (Fig. 3b). Coverage was similar at other difficult regions, although the PCR-free workflow showed improved coverage of a small subset of extreme GC-rich regions.

**Table 3:** Performance of different commercially available library preparation kits for 30X human genome builds

Parameter	Library Preparation Kit					
	Nextera DNA Flex	TruSeq Nano	NEBNext Ultra	Kapa HyperPlus	Kapa HyperPrep	TruSeq DNA PCR-free
No. samples	30	20	4	4	4	6
Includes PCR in protocol	Yes	Yes	Yes	Yes	Yes	No
Fragmentation method	BLT	Sonication	Enzymatic	Enzymatic	Sonication	Sonication
Input direct from blood and saliva	Yes	No	No	No	No	No
Incorporates normalization for inputs ≥100 ng	Yes	No	No	No	No	No
Total assay time, hours	3.5	11	6	6	6	10
Total PF PE reads	3.70E + 08	3.70E + 08	3.70E + 08	3.70E + 08	3.70E + 08	3.70E + 08
Diversity	3.1E + 09	2.0E + 09	1.3E + 09	2.8E + 09	4.6E + 09	1.9E + 09
Autosome coverage at 15X, %	97.9	98.0	97.8	96.1	91.3	98.1
Autosome callability, %	96.7	96.9	96.8	96.6	96.2	96.9
Autosome exon callability, %	98.7	98.4	98.8	98.7	98.5	99.0
SNV recall, %	98.7	98.7	98.8	98.5	97.8	98.8
SNV precision, %	99.8	99.7	99.7	99.1	94.4	99.9
Indel recall, %	94.2	92.9	94.5	93.1	91.0	95.9
Indel precision, %	97.2	94.9	97.7	97.6	96.1	98.3

Libraries were prepared from 100 ng of human gDNA (NA12878) and sequenced on a HiSeqX with 6 samples per flow cell. Data presented is the average of the number of samples indicated for each kit. Data analysis was performed using the BaseSpace Sequence Hub Whole Genome Sequencing 6.0.0 and VCAT 3.0.0 Apps. Callability describes the percentage of base calls in the data set that pass the quality metrics required for making a genotype call; base quality, alignment quality, and minimum coverage levels are considered. Total assay time indicates the time from DNA extraction to library normalization and pooling, with workflow step times determined using specific methods: DNA extraction (QIAamp DNA Mini Kit or Flex Lysis Kit), DNA quantitation (Qubit), DNA fragmentation (Covaris), and manual library normalization and pooling (Bioanalyzer). Calculations assumed that 16 samples were processed at a time with a multichannel pipette. *BLT* Bead-linked transposome, *PE* Paired-end, *PF* Pass filter, *SNV* Single-nucleotide variant, *Indel* Insertion/deletion

Good coverage of difficult regions of the genome suggests that Nextera DNA Flex has significantly reduced bias compared to other library preps, including previous in-solution methods for tagmentation. To further probe this, bacterial genomes with extreme GC content were tested. Small DNA input amounts (0.5, 1, and 10 ng) of three bacterial species of low (*Bacillus cereus* [*B. cereus*]), medium (*Escherichia coli* [*E. coli*]), and high (*Rhodobacter sphaeroides* [*R. sphaeroides*]) GC content were used to prepare libraries with Nextera DNA Flex. The number of PCR cycles was increased as the input amount decreased. Mean fragment sizes measured by Bioanalyzer and median insert size determined by sequencing were consistent between the input amounts for each bacterial species (Table 4). The mean coverage was consistent between the different input amounts for each species, however, the mean coverage was slightly lower for the low-GC *B. cereus* genome than for the other two species. Similarly, variation in coverage across the genome was stable between the different input amounts within each species but varied between the species, with the lowest variability observed for *E. coli* (Table 4).

**Table 4:** Libraries prepared from low DNA inputs of small genomes with low to high GC contents

Organism	Input DNA (ng)	PCR cycles (n)	Library yield (ng/μl)	Median fragment size (bp)	Median insert size (bp)	Mean coverage (X)	CV of coverage across the genome (%)
*B. cereus* (35% GC)	0.5	12	3.2	519	287	52.6	19.1
	1	10	1.9	518	289	53.1	19.2
	10	8	6.3	531	301	52.6	19.2
*E. coli* (51% GC)	0.5	12	5.0	577	304	61.9	16.7
	1	10	3.3	578	310	62.2	16.6
	10	8	8.6	581	308	62.2	16.9
*R. sphaeroides* (69% GC)	0.5	12	6.3	542	299	61.2	21.8
	1	10	3.6	533	301	61.6	21.8
	10	8	10.3	546	294	61.2	21.7

Libraries were prepared from 0.5, 1, or 10 ng bacterial DNA. Data was generated on a HiSeq 2500 Rapid Run (2 × 151 bp) and data analysis performed using BWA aligner application on BaseSpace and SAMtools. Fragment size was determined by Bioanalyzer. Insert size was determined during sequencing. *CV* Coefficient of variation. *GC* Guanine-cytosine

For comparison, we prepared libraries for microbial species of low (*B. cereus*), medium (*E. coli*), and high (*R. sphaeroides*) GC content using five different library preparation kits. In comparison to other kits that showed increased coverage variation for the low and high GC genomes, Nextera DNA Flex coverage was largely unaffected by the genome GC content, with low coverage variability for all three bacterial genomes (Fig. 3c). Improved accuracy in genome assembly for a broad range of microbial organisms with this library preparation kit has been detailed elsewhere [8].

### Preparation of libraries from amplicons, plasmids, and small genomes

**Fig. 4:**
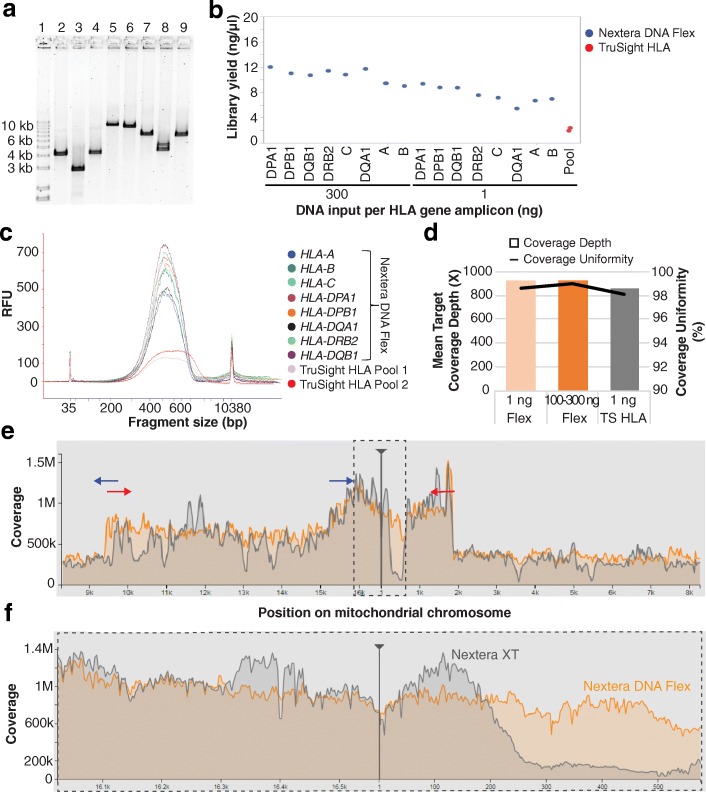
Application of Nextera DNA Flex to human amplicons. **a** Human leukocyte antigen (HLA) gene amplicons used as inputs for library preparation visualized on a 1% agarose gel. Lanes and expected amplicon sizes are as follows: 1, KBL Ladder; 2, *HLA-A* (4.1 kb); 3, *HLA-B* (2.8 kb); 4, *HLA-C* (4.2 kb); 5, *HLA-DPA1* (10.3 kb); 6, *HLA-DPB1* (9.7 kb); 7, *HLA-DQA1* (7.3 kb); 8, *HLA-DRB2* (4.6 kb); 9, *HLA-DQB1* (7.1 kb). **b** Nextera DNA Flex library yields of all HLA amplicons were within the acceptable values of > 4 ng/μl and 9–13 ng/μl for 1 ng and 100–300 ng inputs, respectively. The yields for Nextera DNA Flex libraries were higher than for those prepared using TruSight HLA; for TruSight HLA, libraries were prepared from 1 ng of each amplicon and then pooled. **c** The Bioanalyzer profiles depict library fragment size distributions within the acceptable range; the distribution is narrower for the Nextera DNA Flex libraries (1 ng DNA inputs) than the TruSight HLA libraries. **d** Sequencing coverage depth and uniformity were higher for libraries prepared using Nextera DNA Flex (Flex) compared with TruSight HLA (TS HLA). **e** Libraries were sequenced on a NextSeq 550, with downsampling to 25,000 reads per amplicon. Library preparation using Nextera DNA Flex (orange) resulted in more uniform coverage of the entire human mitochondrial chromosome when compared with Nextera XT (grey). The location of the PCR primers used to create the two mtDNA amplicons are depicted by blue and red arrows. Dotted-line rectangle indicates the D-Loop region. **f** Zoomed in view shows more uniform coverage with Nextera DNA Flex within the D-Loop region

In contrast to purified gDNA, many samples consist of smaller fragments which can present a distinct challenge to library preparation based on enzymatic fragmentation. To test the suitability of Nextera DNA Flex for library preparation from smaller fragments, we evaluated DNA inputs of a range of amplicon sizes. As a test case, eight human leukocyte antigen (HLA) genes were amplified, which ranged in size from 2.8 kb to 10.3 kb (Fig. 4a). Libraries were prepared from 1 ng to 300 ng inputs of each HLA gene amplicon, yielding 120 to 330 ng of sequence-ready library (Fig. 4b). For comparison, libraries were also prepared using the TruSight™ HLA kit v2, which utilizes soluble transposome to produce libraries for ultrahigh resolution sequencing of 11 HLA loci. Because of sensitivity to input amount, only 1 ng of input DNA is suitable for TruSight HLA. Amplicon libraries prepared using Nextera DNA Flex with inputs from 1 to 300 ng showed greater yield (Fig. 4b) and tighter size distribution than TruSight HLA libraries (Fig. 4c, 1 ng input shown). Sequencing revealed better coverage (Fig. 4d) for the Nextera DNA Flex libraries when subsampled to 25,000 reads per amplicon, with comparable mean target coverage and uniformity of coverage.

Libraries were also prepared from two amplicons (9 to 11 kb in size) that when combined spanned the entire mitochondrial genome. For comparison purposes, libraries were also prepared from these amplicons using Nextera XT, a solution-based transposome library preparation kit recommended for use with small genomes and amplicons. The Nextera DNA Flex library provided more uniform coverage of the entire mitochondrial genome (Fig. 4e) and, importantly, better coverage in the non-coding displacement (D) loop region (Fig. 4f), where alterations are present in many cancers [9]. Nextera DNA Flex was also applied to plasmid DNA, and demonstrated similar or better coverage than libraries prepared using Nextera XT (data not shown).

**Fig. 5:**
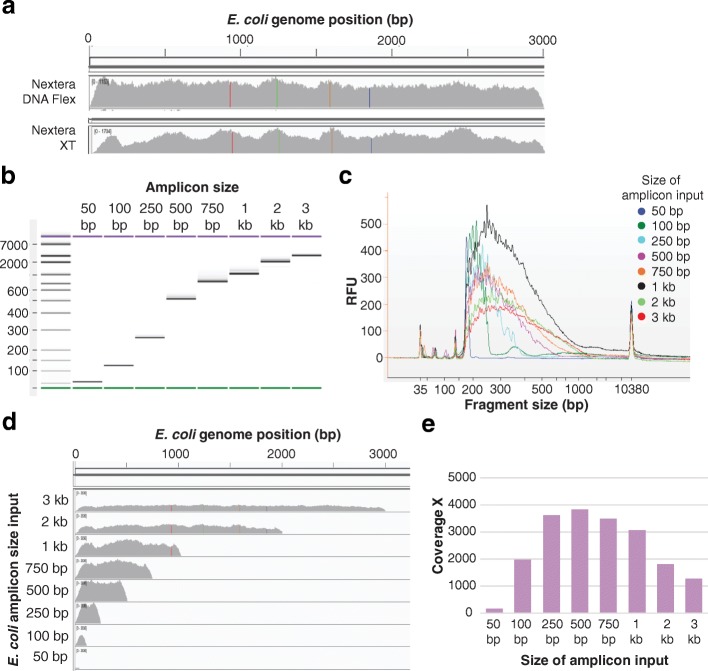
Application of Nextera DNA Flex to bacterial amplicons. **a** Libraries prepared using Nextera DNA Flex showed more consistent, even coverage compared with libraries prepared using Nextera XT; data depicts the sequence coverage of libraries prepared from the 3 kb *E. coli* amplicon. **b** PCR products ranging in size from 50 bp to 3 kb amplified from *E. coli* gDNA visualized on a 1% agarose gel. **c** Libraries prepared from a 1 ng input of these *E. coli* amplicons resulted in Bioanalyzer traces that depicted a slight increase in fragment size with increasing amplicon size. **d** Libraries were sequenced on a MiSeq and coverage of the *E. coli* genome determined for the different amplicon fragment size inputs. Sequenceable libraries were generated from amplicons ranging in size from 50 bp to 3 kb. **e** When sequencing data was downsampled to 25,000 reads, the larger fragment inputs were reaching a coverage maximum

Following the successful preparation of libraries from long-range PCR products, we next evaluated library preparation from smaller amplicons derived from bacterial genomes. Library preparation from a 3 kb *E. coli* amplicon using Nextera DNA Flex resulted in more even coverage than with Nextera XT (Fig. 5). To determine the lower size limit for the use of amplicons, we prepared libraries from varying size (50 bp to 3 kb) PCR amplicons of the same region of the *E.coli* genome (Fig. 5). Due to the small amplicon length, we altered our size selection, opting for a single 1.8× volume addition of SPBs instead of the standard double-sided size selection (see methods). Due to the single step size selection, the fragment size profiles for these *E. coli* amplicon libraries (Fig. 5) showed the expected broadening in the fragment peak and increase in the median fragment size with increasing amplicon size. Libraries were successfully generated from all amplicon sizes (50 bp to 3 kb). At a subsampling of 25,000 reads per amplicon, the larger fragments (> 500 bp) reached a coverage maximum (Fig. 5). The coverage obtained for different amplicon size inputs is shown in Fig. 5. Interestingly, comparable data were generated using our standard protocol, with double-sided size selection (data not shown). Overall, although libraries were generated and sequenced from amplicons as small as 50 bp, reduced efficiency of library generation was observed at smaller amplicon sizes, with a significant drop in coverage below 250 bp, and a further significant drop below 100 bp.

### Preparation of libraries from FFPE samples

**Fig. 6:**
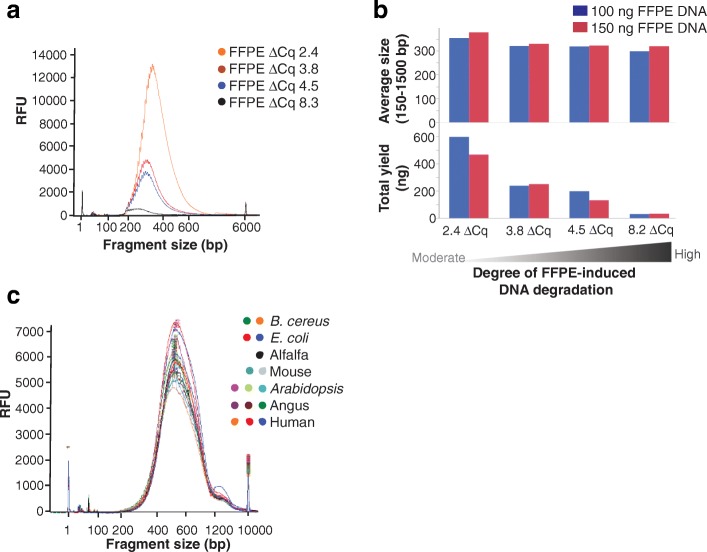
Bioanalyzer traces of libraries prepared from various sample types and species. **a** Libraries prepared from samples with a varied degree of formalin fixation; a higher ΔCq indicates more FFPE-induced DNA degradation compared with a positive control. **b** Increasing FFPE-induced DNA degradation has a small effect on average fragment size but a marked effect on the total library yield. Increasing the DNA input from 100 ng to 150 ng did not increase library yield, indicating bead saturation at a DNA input of around 100 ng regardless of the degree of DNA degradation. **c** Libraries prepared from gDNA from a range of animal (human, Angus, and mouse), plant (Arabidopsis and alfalfa), and bacterial (*E. coli* and *B. cereus*) species

The results of the amplicon length study suggest that the Nextera DNA Flex workflow has the potential to generate libraries from degraded samples. One of the key potential application areas for whole-genome sequencing (WGS) is cancer genomics research. One of the challenges in this area is generating libraries that can be sequenced from degraded DNA samples resulting from formalin fixation. To explore the capacity of Nextera DNA Flex to generate libraries from formalin-fixed paraffin-embedded (FFPE) samples, we tested DNA extracted from four independent FFPE preparations with moderate (ΔCq 2.4) to very severe (ΔCq 8.2) degradation. Due to the degraded nature of the samples, we made three adjustments to the workflow: first, the DNA was ‘repaired’ with a FFPE repair kit to improve DNA quality (remove damaged bases and reduce the level of single stranded DNA); second, we increased the number of PCR cycles to improve yield; third, because we expected smaller fragments, we used a single-sided SPRI size selection to increase yield and purify the smaller fragments expected from tagmentation of degraded DNA. In all cases, libraries were generated from 100 ng input DNA in sufficient amounts for sequencing (Fig. 6a and b), however, progressively lower yields were observed with increased degradation (Fig. 6b). Based on a FFPE qPCRbased quality control assay (Illumina, cat. no. WG-321-1001), we estimate that more than 99% of the target gene in the FFPE ΔCq 8.2 sample was unable to be amplified. Nevertheless, we still obtained sufficient amounts (30 μl at 4.8 nM) of library for sequencing on a HiSeq X sequencer. The remaining three FFPE samples represented significantly degraded DNA (ΔCqs of 2.4, 3.8, and 4.5; estimated 80–95% of control target degraded), but still produced library yields (29 to 80 nM) comparable to high quality gDNA prepared using the standard Nextera DNA Flex workflow. Libraries were sequenced on a HiSeq X, resulting in 84% to 96% of reads aligned to human genomes with median fragment lengths of 84 to 270 bp and library diversity of greater than 1.4 billion molecules for all samples. We conclude that Nextera DNA Flex, with minor workflow modifications, generates sequence-ready libraries from DNA extracted from FFPE samples with various levels of degradation. Typically, the BLT beads saturate and yields normalize with 100 ng or more of input DNA. We wondered if with degraded DNA, the saturation point might change, so we tested the four FFPE samples at 100 ng and 150 ng inputs. Interestingly, although overall yields differed depending on DNA quality, we observed no further increase in yield with 150 ng input compared to 100 ng input DNA (Fig. 6b), indicating that the beads were still saturating near 100 ng of DNA input regardless of the degradation state of the DNA.

### Broad applicability

Nextera DNA Flex was designed to be flexible in terms of genome size, sample type, and input amount. Preparation of libraries from a variety of mammalian, plant, and microbial species revealed Bioanalyzer traces with consistent fragment size and concentrations of libraries between species (Fig. 6c).

The workflow improvements described make Nextera DNA Flex particularly well-suited to automation for high-throughput applications. Libraries prepared on the liquid handling platforms produced comparable sequencing metrics (eg, median insert size, coverage, duplicates, diversity) to manual preparations (Table 5).

**Table 5:** Libraries prepared from human gDNA by manual preparation and two automated liquid handler platforms

Metrics		Manual Preparation	Hamilton NGS STAR™	Eppendorf ep*Motion*® 5075 t
Yield (Qubit), ng/μl	Run 1 Run 2	12.4–14.1 11.1–13.5	9.1–11.1 13.7–15.6	12.8–14.2 14.1–17.4
Yield CV, %	Run 1 Run 2	5.1 6.2	8.3 4.8	3.5 7.3
Index CV, %	Run 1 Run 2	10.8 12.1	13.7 11.7	11.0 12.6
Median insert size, bp (optimal: 350 ± 50 bp)	Run 1 Run 2	348–357 350–363	375–391 377–385	335–344 351–368
Autosome mean coverage, X		30–32	30–32	30–32
Coverage across the human genome at 15X, %		97.6	97.7	97.4
Coverage across exonic regions at 10X, %		99.6	99.6	99.8
Mean diversity		> 2.0e^9^	> 2.0e^9^	> 2.0e^9^
Autosome Callability, %		95.0	94.5	94.5

Libraries were prepared from 200 to 300 ng of human DNA (NA12878) using an Illumina Qualified method. Data is presented for two 8-plex runs on a HiSeqX system, with sequencing reads trimmed to a 30X depth (380 million reads, 2 × 151 bp). Data analysis performed using the BaseSpace Sequence Hub Whole Genome Sequencing v5.0 App. *CV* Coefficient of variation

## Discussion

We have demonstrated that utilization of a bead-linked transposome technology enables fast library preparation for a wide range of applications. The incorporation of on-bead tagmentation reduces hands-on time, allowing libraries to be prepared within a few hours.

One of the limitations of other DNA library preparation methods, including solution-based tagmentation technology, is non-uniform coverage in heterochromatic or GC-rich DNA regions [10]. In coupling transposomes to beads, we focused on optimizing the library preparation protocol for applicability across a range of input amounts and sample types. An additional desirable property that emerged from immobilizing the transposomes on beads was improved coverage uniformity at difficult regions. As a result, good recall and precision was achieved for human variant calling; reducing the number of PCR cycles and using updated analytics can further improve nucleotide variant detection.

The Nextera DNA Flex workflow was optimized for use across a range of gDNA input amounts, ensuring an adequate yield and a fragment size profile suitable for WGS. An average fragment size of around 350 bp combined with a tight size profile helps to minimize clipped bases in WGS. In contrast, other library preparation kits do not achieve consistent insert sizes across different DNA input amounts, which can lead to increased soft clipping and an increased sequencing requirement. Furthermore, we describe methods to achieve different insert sizes if desired for other applications by altering the size selection protocol used during post-amplification cleanup. Small targets, for example, can present a challenge for enzymatic fragmentation. Here, evaluation of the amplicon cliff edge revealed that libraries can be prepared from amplicons as small as 50 bp using this method, albeit with a significant drop in efficiency below 250 bp. In comparison to a solution-based transposome method, this bead-based methodology offered improved coverage for amplicon libraries. In addition, we successfully prepared libraries from degraded DNA samples resulting from formalin fixation. As the quality of DNA isolated from FFPE samples is variable, DNA quantitation is recommended prior to library preparation. Further optimization could be directed to integrating and optimizing FFPE repair prior to library preparation and optimizing size selection for targets with levels of degradation relevant to specific user needs.

One of the key features of this technology is the consistency that it brings to DNA library preparation. We showed that the beads become saturated at DNA inputs above approximately 100 ng, which results in normalization of library yields, eliminating the need for quantitation of input material and individual libraries for many applications. This is expected to offer both a significant cost and time saving for high-throughput applications, such as population-level human WGS studies. An additional advantage for high-throughput applications with this library preparation kit is compatibility with automation, with libraries prepared using liquid handling systems equal in quality to those prepared manually. A limitation of previous transposome-based methods is inconsistent insert size, which can be influenced by the ratio of enzyme to target DNA. Here, the use of bead-linked transposomes resulted in a consistent insert size distribution across a broad range of DNA inputs and improved coverage of genomes and regions with extreme GC composition.

## Conclusion

In summary, we have shown that this methodology offers broad applicability, supporting a wide spectrum of DNA input ranges as well as integrated extraction of blood and saliva samples. With other methods, a requirement for large input amounts can be a limiting factor. Using this method, libraries were prepared from DNA inputs ranging across five orders of magnitude (10 pg to 1 μg). Moreover, we have demonstrated application to a variety of sample types, from small genomes and amplicons to large and complex genomes. This methodology generates normalized libraries for sequencing to facilitate a quantification-free workflow, and expands the range of possible sample types, providing significant improvements in flexibility and performance over solution-based library preparation kits.

## Methods

### Samples

Genomic DNA samples were obtained from the following sources: Human (Coriell Institute for Medical Research, cat. no. NA12878), Angus (*Bos taurus*; Angus blood obtained from MB Genetics, Inc. and extracted with MasterPure-Blood kit from Epicentre [an Illumina company]), Mouse (Promega, cat. no. G309A), Arabidopsis (BioChain Institute, cat. no. D1634310–5), Alfalfa (*Medicago sativa* obtained from Mountain Rose Herbs, cat. no. ALF-P10Z; DNA extracted with MasterPure kit from Epicentre), *E. coli* (ATCC, cat. no. 700926D-5), *B. cereus* (ATCC, cat. no. 10987), and *R. sphaeroides* (ATCC, cat. no. 17023 [strain ATH 2. 4. 1]), formalin treated quantitative multiplex reference standards (Horizon, cat. Nos. HD798, HD799, HD803, HD729 Tier 5 [Matched control]), FFPE donor samples (ProteoGenex, cat. Nos. 081454 T2(1), 017102 T2(3), 018217 T2(2), and 033014 T2(3)). Human blood and saliva samples were collected from healthy volunteers that were aged > 18 years and provided written consent to use of their samples for research under an approved Institutional Review Board (IRB) protocol; volunteers were recruited from Illumina, Inc. through IRB-approved posters describing the study that were posted on in-house bulletin boards.

### Nextera DNA flex library preparation standard protocol

**Fig. 7:**
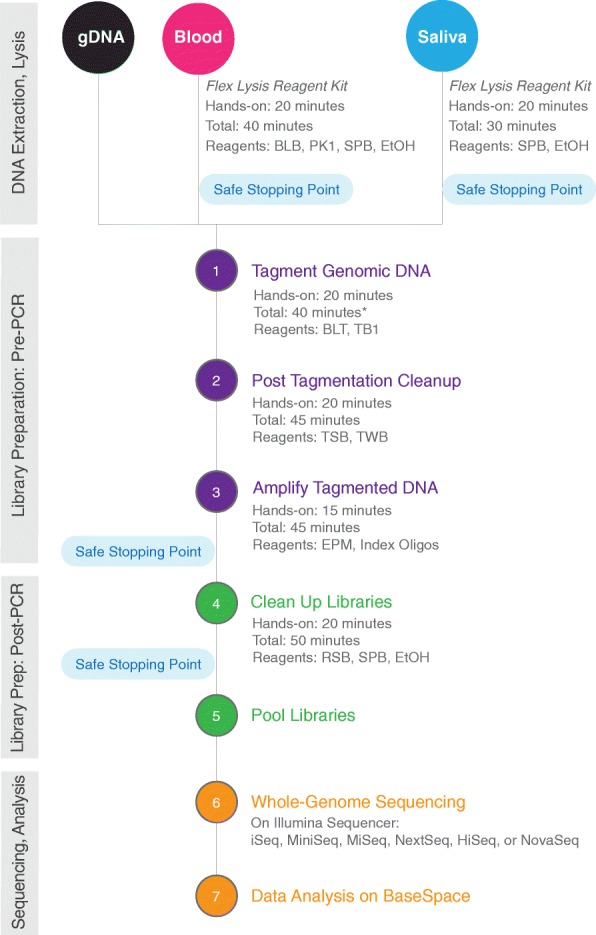
Nextera DNA Flex Library Prep workflow overview. * Time estimates based on preparing 16 samples using a multichannel pipette. BLB, blood lysis buffer. BLT, bead-linked transposome. EPM, enhanced PCR mix. EtOH, Ethanol. PK1, proteinase K. RSB, resuspension buffer. SPB, sample purification beads. TB1, tagmentation buffer 1. TSB, tagment stop buffer. TWB, tagment wash buffer

Unless otherwise specified, all libraries were prepared using the following standard protocol as described in the manufacturer’s instructions (Illumina, Nextera DNA Flex Library Prep Reference Guide). All reagents listed below are included in the Nextera DNA Flex kit (Illumina, cat. Nos. 20,018,704, 20,018,705). An overview of the workflow is depicted in Fig. 7. For each sample, between 2 and 30 μl of DNA (1–500 ng) was loaded into one well of a 96-well PCR plate, with nuclease-free water added to bring the volume up to a total of 30 μl. Then 20 μl of well-mixed tagmentation master mix (1:1 mixture of Bead-Linked Transposome and Tagmentation Buffer) was added to bring the final volume up to 50 μl. After all samples were loaded, the plate was sealed and incubated at 55 °C for 15 min.

After completion of the tagmentation reaction, post tagmentation cleanup was done by the addition of 10 μl of Tagment Stop Buffer to each reaction and resuspension of the beads. The plate was sealed, incubated at 37 °C for 15 min in a thermal cycler, and then placed on a 96-well plate magnet (Thermo Fisher Scientific, cat. no. AM10027) for 3 min (or until the solution was clear). The supernatant was discarded. Next, two rounds of washes were performed, with each round involving the following steps: the plate was removed from the magnet, 100 μl of Tagment Wash Buffer was added, the plate was placed back on the plate magnet for 3 min (or until clear), and the supernatant discarded. The washed beads were then resuspended in 100 μl of Tagment Wash Buffer before the plate was placed on the magnet for a further 3 min (or until clear).

The third part of the protocol was amplification of the tagmented DNA using a limited-cycle PCR reaction. For each reaction, 40 μl of PCR master mix was made by mixing 20 μl of Enhanced PCR Mix with 20 μl of nuclease-free water. The Tagment Wash Buffer was completely removed from each sample well prior to removal of the plate from the magnet. Then 40 μl of PCR master mix was added to each sample, 10 μl of index adapters were added according to the index kit configuration being used (for 24 plex [single index] or 96 plex [dual index], 10 μl of primer mix was used; for 24 plex with dual index, 5 μl i5 adapter and 5 μl i7 adapter was used), and the sample was mixed by pipetting 10 times. The plate was sealed and placed in a thermal cycler with a heated lid, and run using the following PCR parameters: 68 °C for 3 min, 98 °C for 3 min, then 5 cycles of 45 s at 98 °C, 30 s at 62 °C, and 2 min at 68 °C, before a final minute at 68 °C; five PCR cycles was used unless otherwise specified. The plate was then centrifuged for 1 min at 280 x g.

The next step in the DNA library preparation involved cleanup of the amplified libraries, which was carried out using a double-sided bead purification procedure, using SPRI beads (termed Sample Purification Beads, SPBs). Note that the SPBs are different beads to those used in the tagmentation step above. The PCR plate was placed on a plate magnet for 5 min. Next, 45 μl of the clear supernatant was transferred to a fresh midi plate. For each sample, a diluted solution of SPBs was prepared by mixing 45 μl of SPBs with 40 μl of nuclease-free water. The entire 85 μl solution was added to each sample well of the fresh midi plate containing the 45 μl of supernatant, and the sample was mixed by pipetting 10 times. The plate was incubated at room temperature for 5 min, then placed on a plate magnet for a further 5 min (or until clear). During incubation, 15 μl of the undiluted SPBs was added to each well of a second, fresh midi plate. For each sample, 125 μl of supernatant from the first midi plate was transferred to the second midi plate containing the 15 μl undiluted SPBs and the sample mixed by pipetting 10 times. The plate was incubated at room temperature for 5 min then placed on a plate magnet for 5 min. The supernatant was discarded and 200 μl of 80% ethanol was added to the plate on the magnet followed by a 30 s incubation. The ethanol was removed and the beads were washed again with 80% ethanol before being allowed to dry on the plate magnet for 5 min to ensure complete removal of ethanol. The plate was removed from the magnet and 32 μl of Resuspension Buffer was added to the beads. The beads were resuspended and incubated at room temperature for 2 min. The midi plate was then placed on the plate magnet for 2 min before 30 μl of the supernatant containing the DNA library was transferred to a fresh 96-well plate.

The last step required pooling of the DNA libraries. For DNA inputs of 100 to 500 ng, libraries were pooled by volume (5 μl per sample; up to 96 samples) into a 1.5 mL tube prior to sequencing. The single pooled library was quantified using Qubit or PicoGreen and diluted to 2 to 4 nM with Resuspension Buffer; the required molarity varies by sequencer. For DNA inputs of less than 100 ng, each library was quantified and then diluted to the required molarity with Resuspension Buffer and 10 μl of each diluted sample mixed in a single tube. The pooled libraries were then run on an Illumina sequencer (ie, MiniSeq, MiSeq, NextSeq 550, HiSeq2500, HiSeqX, or NovaSeq).

Where described, library quality was determined by running 1 μl of the pooled library or an individual library on a Bioanalyzer (Agilent 2100 Bioanalyzer) using a High Sensitivity DNA kit (Agilent, cat. no. 5067–4626) or on a Fragment Analyzer (Advanced Analytical Fragment Analyzer) with the High Sensitivity NGS Fragment Analysis Kit (Advanced Analytical, cat. no. DNF-474).

### Integrated extraction protocol for blood and saliva

An integrated extraction protocol for blood or saliva was developed that is designed for use with the DNA Flex library preparation kit. For the preparation and lysis of blood samples, fresh whole blood was processed using the Flex Lysis Reagent kit (Illumina, cat. no. 20015884). Fresh whole blood was collected into EDTA collection tubes and stored at 4 °C before processing. A lysis master mix was prepared by mixing the following volumes for each sample: 7 μl of Blood Lysis Buffer, 2 μl of proteinase K, and 31 μl of nuclease-free water. For each sample, 10 μl of blood, 40 μl of the lysis master mix, and 20 μl of SPBs was added to one well of a 96-well PCR plate and mixed by pipetting the solution 10 times. The plate was sealed and incubated for 10 min at 56 °C on a thermal cycler with a heated lid. The plate was then placed on a plate magnet for 5 min, the supernatant was discarded, and 150 μl of 80% ethanol was added. After incubation for 30 s on the magnet, the ethanol was discarded, and the plate was removed from the magnet. The beads were resuspended in 30 μl of water and ready for library preparation.

Saliva was collected in Oragene DNA Saliva Collection tubes (DNA Genotek, cat. Nos. OGR-500, OGD-510), which were incubated for at least 1 h at 50 °C to lyse the cells before thorough mixing by vortexing. For each sample, 20 μl of water and 30 μl of saliva was added to one well of a 96-well PCR plate and slowly mixed by pipetting. Then 20 μl of SPBs was added to the sample well and the beads were thoroughly mixed by pipetting the solution 10 times. The plate was incubated for 5 min at room temperature before being placed on a plate magnet for 5 min. The supernatant was removed and 150 μl of 80% ethanol was added to the bead pellet. The plate was then allowed to stand for 30 s on the magnet before removal of the ethanol and then removal of the plate from the magnet. The beads were resuspended in 30 μl of water and ready for library preparation.

### DNA input amount and normalization

The impact of DNA input amount on the yield and insert size was evaluated by preparing libraries from 1 ng, 10 ng, 25 ng, 50 ng, 100 ng, 250 ng, 500 ng, or 1 μg of NA12878. The library yields were compared for the different input amounts to determine at what input threshold the beads became saturated. The effect of input amount on median insert size was determined by sequencing the libraries on a MiSeq.

Additional experiments were done to evaluate libraries prepared from low DNA input amounts. Libraries were prepared using 0.01 ng to 100 ng of NA12878. The standard protocol was followed for all libraries, with the exception that for less than 1 ng input, one PCR cycle was added for each halving of the input amount. Each library was loaded on one lane of a HiSeq X and sequenced with 2 × 150 paired-end reads. Samples were analyzed using the BaseSpace™ Whole Genome Sequencing app and the Variant Calling Assessment Tool 3.0.0 (Illumina).

### Impact of modifying library preparation conditions

The impact of PCR conditions on callability was also assessed by preparing libraries from 100 ng of NA12878 following the standard protocol but using 2, 3, 4, or 5 PCR cycles. Callability describes the percentage of base calls in the data set that pass the quality metrics required for making a genotype call; base quality, alignment quality, and minimum coverage levels are considered.

In the process of evaluating the optimal conditions for post-amplification cleanup, the impact of modifying the cleanup conditions on the library and resultant sequencing parameters was assessed. The standard library protocol described above was used with modification of the post-amplification cleanup protocol. The DNA input for these experiments was 100 ng. First, a single SPB cleanup (0.7X bead concentration) was compared with a double SPB cleanup (0.5X bead concentration in the first cleanup step, and 0.7X bead concentration in the second cleanup step). For this experiment, the impact on the library (insert size profile, median insert size) and sequencing parameters (clipped bases) was assessed. Second, the bead volume input was varied in either the first (22–67 μl; the 40 μl volume of nuclease-free water used to dilute the beads was fixed) or second (8–24 μl) cleanup, keeping the second and first bead volumes fixed, respectively. Libraries were analyzed on a Bioanalyzer to determine the median library fragment size and the DNA yield was determined by Qubit.

### Library preparation from amplicons, plasmid, and small genomes

Using NA12878 as a source, amplicons were generated for human leukocyte antigen (HLA) genes: *HLA-A* (4.1 kb), *HLA-B* (2.8 kb), *HLA-C* (4.2 kb), *HLA-DPA1* (10.3 kb), *HLA-DPB1* (9.7 kb), *HLA-DQA1* (7.3 kb), *HLA-DRB2* (4.6 kb), and *HLA-DQB1* (7.1 kb). Amplicons were prepared according to the recommended protocol for the TruSight HLA v2 Sequencing Panel (Illumina, cat. no. 20007429). After amplification, PCR products were purified in bulk with a 0.7X concentration of SPBs before running 50 ng of each on a 1% agarose gel. Nextera DNA Flex libraries were prepared from 1 ng and 100 to 300 ng inputs of each amplicon; 100 ng inputs were used for HLA-B and DPA1, 300 ng inputs were used for all other amplicons. TruSight HLA library pools were prepared using the TruSight HLA v2 Sequencing Panel (Illumina, cat. no. 20000215): 1 ng of each amplicon was individually tagmented, 10 μl of each tagmented amplicon was pooled before cleanup with SPBs; the manufacturer’s recommended protocol was followed except at the Normalize HLA PCR Amplicons step, where the two amplicon pools were manually quantitated to maintain consistency with the Nextera DNA Flex protocol. The fragment size profiles and yields of the libraries were determined, then libraries were sequenced on a NextSeq and coverage determined when downsampled to 25,000 reads per amplicon.

Human mitochondrial DNA (mtDNA) amplicons were prepared from NA12878 using the Human mtDNA Genome Guide (Illumina, Human mtDNA Genome Guide [15,037,958 01]). As per this protocol, two primer pairs were used to generate mtDNA amplicons of 9.1 kb and 11.2 kb. Both amplicons cover the non-coding displacement (D) loop region; coverage of this region is important as alterations in this region are present in many cancers. Libraries were prepared from 1 ng and 100 ng inputs of both amplicons using Nextera DNA Flex kit and from a 1 ng input using the Nextera XT DNA Library Preparation kit (Illumina, cat. Nos. FC-131-1024, FC-131-1096). Libraries were sequenced on a NextSeq™ 550 and subsampled to 25,000 reads per sample. Coverage between the different library preparation kits were compared across the entire mitochondrial genome.

Nextera DNA Flex libraries were prepared using 1 ng inputs of *E. coli* PCR products of varying sizes (50 bp – 3 kb). The fragment size profiles of the libraries were determined using a Bioanalyzer and the libraries sequenced on a MiSeq to determine coverage when downsampled to 25,000 reads per sample. “Downsampled” indicates that the total sequence reads for a given sample were reduced to the specified value using the BaseSpace App FASTQ 2.2.0, with the app randomly picking the specified number of reads; this facilitates comparison of coverage between samples as each downsampled sample has the same number of reads. A library was also prepared from a 3 kb amplicon of *E. coli* using Nextera XT following manufacturer’s recommendations. Coverage of the *E. coli* genomes generated from Nextera DNA Flex and Nextera XT 3 kb amplicon libraries were compared.

The suitability of the Nextera DNA Flex kit for small genomes was evaluated using three bacterial species of low (*B. cereus*, 35%), medium (*E. coli*, 51%), and high (*R. sphaeroides*, 69%) GC content. Libraries were prepared from 0.5 ng, 1 ng, and 10 ng of gDNA from each species using 12, 10, and 8 PCR cycles, respectively, for the different DNA inputs. The library yields, fragment size, insert size, and coverage were determined.

### Application to varied species and sample types

The broad applicability of the Nextera DNA Flex kit was determined through the application to a range of species with small to large/complex genomes and a variety of sample types. Libraries were prepared from 100 ng inputs of human (NA12878), Angus, Arabidopsis, mouse, alfalfa, *E. coli*, and *B. cereus* gDNA. The fragment size profile of each library was determined by a Fragment Analyzer.

We evaluated the potential to generate libraries from poor-quality samples. This involved preparation of libraries from 100 ng of mild (Horizon, cat. no. HD798), moderate (Horizon, cat. no. HD799), or severe (Horizon, cat. no. HD803) formalin treated quantitative multiplex reference standards as well as the matched control (Horizon, cat. no. HD729 Tier 5). Due to the degraded nature of the sample, we made three adjustments to the workflow: first, the DNA was ‘repaired’ with a standard FFPE repair kit (Illumina, cat. no. WG-321-1002) to improve DNA quality; second, we increased the number of PCR cycles from 5 to 8 to improve yield; third, because we expect smaller fragments, we used a single-sided SPRI size selection with 1.8× volume of SPBs to increase yield and purify the smaller fragments expected from tagmentation of degraded DNA. With increasing fixation, there was a drop in the library yield and a reduction in the fragment size. However, despite the challenges of degraded DNA, the protocol modifications permitted increased yield (~ 150–400 nM, 24–110 ng/μl) relative to the standard protocol, and in all cases, the libraries were generated in sufficient quantity for multiple HiSeq X runs. Libraries were also prepared from FFPE donor samples following the protocol above.

### Comparison of different library preparation kits

The performance of library preparation for large genomes was compared between Nextera DNA Flex and a selection of other commercially available library preparation kits. Library preparation kits from Illumina used in comparisons to Nextera DNA Flex included: Nextera DNA, TruSeq Nano (Illumina, cat. Nos. FC-121-4001, FC-121-4002, FC-121-4003), and TruSeq DNA PCR-free (Illumina, cat. Nos. FC-121-3001, FC-121-3002, FC-121-3003). Third-party comparator kits included NEBNext Ultra DNA Library Prep Kit (New England BioLabs, cat. no. E7370S), Kapa HyperPrep Kits (Roche, cat. Nos. KK8502 [with library amplification], KK8503 [without amplification module]), Kapa HyperPlus Kits (Roche, cat. Nos. KK8512 [with library amplification], KK8513 [PCR-free]). Libraries were prepared from 100 ng NA12878 following the manufacturers’ instructions for each kit, and sequenced on a HiSeq X with six samples per flow cell. Analysis of the 30X human genome builds was performed using two BaseSpace Apps, WGS 6.0.0 followed by VCAT 3.0.0 (Illumina).

### Sequencing analysis

In the experiments described here, a variety of Illumina sequencers were used to demonstrate the compatibility of this library preparation kit with all Illumina sequencers; the choice of sequencer to be used in a given experiment was determined based on the number and type of samples and the desired sequencing depth. BaseSpace applications used for human genome build analysis were Whole Genome Sequencing v6.0.0, Whole Genome Sequencing v5.0.0, Variant Calling Assessment Tool v3.0.0, FASTQ Toolkit v2.2.0, and Integrated Genomics Viewer v2.1.2. BaseSpace applications used for small genome and amplicon analysis were BWA aligner v1.1.4, SPADEs genome assembler v3.9.0, Enrichment v3.0.0, mtDNA Variant Processor v1.0.0, mtDNA Variant Analyzer v1.0.0, and Integrated Genomics Viewer v2.1.2.

Determination of coverage uniformity involved evaluation of coverage at bias motifs known to encapsulate common sources of coverage bias [10]: low GC, high GC, huge GC, high AT, huge AT, AT repeats, and G or C rich regions. For regions of at least 200 bp, extremes of nucleotide content were assigned when the middle 100 bases could be classified as follows: “low”, 10% or less of the specified nucleotides; “high”, 75% or more of the specified nucleotides; “huge”, 85% or more of the specified nucleotides; eg, high GC content indicates at least 75% GC content within the central 100 bp. “at_dinucleotides” indicated 130 base regions in which the middle 30 bases were repeated AT dinucleotides. “G rich” or “C rich” indicated 130-base regions in which the middle 30 bases contained at least 80% Gs or 80% Cs, respectively.

### Automation of library preparation for high throughput applications

The Nextera DNA Flex library preparation kit was tested on a variety of liquid handling platforms. Libraries were prepared using 200 to 300 ng of human gDNA. The resulting libraries were sequenced to a 30X depth on a HiSeqX system. Libraries were prepared on NGS STAR (Hamilton), epMotion 5075 t (Eppendorf), Biomek i7 (Beckman), Freedom Evo (Tecan), Sciclone (PerkinElmer), and Bravo NGS (Agilent) systems; with the exception of Agilent, all other listed providers are Illumina Automation Partners. Data were generated during testing of automated methods to achieve Illumina Qualified designation.
